# Evaluation of 3D MRI for early detection of bone edema associated with apical periodontitis

**DOI:** 10.1007/s00784-023-05159-z

**Published:** 2023-07-18

**Authors:** Georg C. Feuerriegel, Egon Burian, Nico Sollmann, Yannik Leonhardt, Gintare Burian, Magdalena Griesbauer, Caspar Bumm, Marcus R. Makowski, Monika Probst, Florian A. Probst, Dimitrios C. Karampinos, Matthias Folwaczny

**Affiliations:** 1 Department of Diagnostic and Interventional Radiology, Klinikum rechts der Isar, School of Medicine, Technical University of Munich, Ismaninger Strasse 22, 81675 Munich, Germany; 2 Department of Diagnostic and Interventional Neuroradiology, Klinikum rechts der Isar, School of Medicine, Technical University of Munich, Munich, Germany; 3 Department of Diagnostic and Interventional Radiology, University Hospital Ulm, Ulm, Germany; 4 TUM-Neuroimaging Center, Klinikum rechts der Isar, Technical University of Munich, Munich, Germany; 5 Department of Prosthodontics, LMU University Hospital, Ludwig-Maximilians-University, Munich, Germany; 6 Department of Restorative Dentistry and Periodontology, LMU University Hospital, Ludwig-Maximilians-University, Munich, Germany

**Keywords:** Magnetic resonance imaging, Periodontal disease, Root canal, Periapical osteolysis

## Abstract

**Objectives:**

To detect and evaluate early signs of apical periodontitis using MRI based on a 3D short-tau-inversion-recovery (STIR) sequence compared to conventional panoramic radiography (OPT) and periapical radiographs in patients with apical periodontitis.

**Materials and methods:**

Patients with clinical evidence of periodontal disease were enrolled prospectively and received OPT as well as MRI of the viscerocranium including a 3D-STIR sequence. The MRI sequences were assessed for the occurrence and extent of bone changes associated with apical periodontitis including bone edema, periradicular cysts, and dental granulomas. OPTs and intraoral periapical radiographs, if available, were assessed for corresponding periapical radiolucencies using the periapical index (PAI).

**Results:**

In total, 232 teeth of 37 patients (mean age 62±13.9 years, 18 women) were assessed. In 69 cases reactive bone edema was detected on MRI with corresponding radiolucency according to OPT. In 105 cases edema was detected without corresponding radiolucency on OPT. The overall extent of edema measured on MRI was significantly larger compared to the radiolucency on OPT (mean: STIR 2.4±1.4 mm, dental radiograph 1.3±1.2 mm, OPT 0.8±1.1 mm, *P*=0.01). The overall PAI score was significantly higher on MRI compared to OPT (mean PAI: STIR 1.9±0.7, dental radiograph 1.3±0.5, OPT 1.2±0.7, *P*=0.02).

**Conclusion:**

Early detection and assessment of bone changes of apical periodontitis using MRI was feasible while the extent of bone edema measured on MRI exceeded the radiolucencies measured on OPT.

**Clinical relevance:**

In clinical routine, dental MRI might be useful for early detection and assessment of apical periodontitis before irreversible bone loss is detected on conventional panoramic and intraoral periapical radiographs.

## Introduction

Apical periodontitis (AP) is an inflammatory process caused by pathogens and their toxins occupying the root canal system, and it is commonly initiated by either trauma, caries, or tooth wear [1, 2]. It starts with a non-specific inflammatory reaction in the periapical region of an infected tooth, followed by a specific innate and adaptive immunological response [3]. The complex interaction of inflammatory cells with numerous biochemical mediators, microbial products, and toxins finally establishes the periradicular pathosis, which can lead to periapical bone resorption and distinctive radiolucencies seen on dental radiography [2, 3]. Moreover, AP mostly presents either as an acute/symptomatic or chronic/asymptomatic disease and histopathologically, the majority of apical periodontitis can be classified into periapical cysts, granulomas, or periapical abscesses, which all appear radiolucent on periapical radiographs [4, 5]. Early treatment of AP is essential as untreated infections are associated with loss of teeth and reduced quality of life as well as, in severe cases, with cellulitis and osteomyelitis [6, 7]. Furthermore, undetected AP constitutes a permanent low-grade infection and is associated with increased risk for cardiovascular diseases [7]. With an individual prevalence of up to 52%, which rises up to 62% for patients over the age of 60 years, AP represents a widespread and most likely underestimated issue with relevant socioeconomic burden due to the high treatment costs [1, 8].

The most established treatment for AP includes root canal treatment, which generally shows good outcome but failure rates of up to 14–16% have been reported, representing a large number in regard to the high prevalence, making prevention as well as early detection highly necessary [2, 9]. So far, the diagnostic approach for AP includes a thorough anamnesis, clinical examination, and radiographic evaluation either using dental radiography, oral panoramic radiography (OPT), or cone beam computed tomography (CBCT) [10]. Conventional radiography offers appropriate sensitivity regarding the detection of periapical radiolucency but only low specificity compared to CBCT, which in turn offers a high sensitivity and specificity but with the downside of exposing the patient to ionizing radiation [11].

In clinical radiology, magnetic resonance imaging (MRI) has been used for more than 20 years for detection of acute and chronic inflammatory changes in bones, musculature, and the gastrointestinal system [12, 13]. Recently, literature on the detection of bone edema using MRI to generate markers for subtle inflammatory intraosseous changes in the alveolar bone but also in the gingiva is increasing [14–19]. Probst et al. showed that intraosseous edema is strongly associated with the severity of inflammatory activity in generalized periodontitis, mirrored by clinical parameters like bleeding on probing or attachment loss [20]. Integrating MRI in the clinical workflow not only for detection of periodontitis but also for clinically silent apical inflammation in the form of circumscribed bone edema would be another step towards dental primary prevention.

Therefore, the aim of this study was to detect and evaluate early signs of AP with edematous changes within the alveolar bone using 3T MRI based on a three-dimensional (3D) short tau inversion recovery (STIR) sequence, and to compare findings with conventional OPT and periapical radiographs in patients with periodontal disease. Our goal was to review the potential of MRI for the detection of early inflammatory processes in the bone. The null hypothesis was that MRI could not generate additional findings regarding the severity and extent of periapical changes in asymptomatic patients compared to radiation-based conventional imaging.

## Materials and methods

### Patient selection

Patients with clinical evidence of periodontal disease who presented at the Department of Periodontology, Ludwig-Maximilians-University Munich, were enrolled prospectively between March 2018 and April 2019. In total, 232 teeth of 37 patients (mean age 62 ± 13.9 years, 18 women) were assessed. All patients were clinically evaluated by dentists. Clinical findings were not available to the MRI examiners, nor were the results of MRI available to clinical examiners.

The study was conducted according to the STROBE guidelines for observational studies [21]. All procedures were conducted according to the principles expressed in the Declaration of Helsinki. Written patient consent was obtained. The prospective analysis was approved by our institutional review boards (Technical University of Munich: Ref.-No.185/18 S and Ludwig-Maximilians-University Munich: Ref.-No. 18-657). The study was retrospectively registered at the DRKS (German Clinical Trials Register, DRKS00020761).

### MRI acquisition

All subjects were examined using a 3T MRI scanner (Ingenia; Philips Healthcare, Best, The Netherlands) with a dedicated 16-channel head-neck and spine coil (dStream Head Neck Spine coil, Philips Healthcare, Best, The Netherlands). The following sequences were acquired: (1) a 3D STIR sequence with the following parameters: echo time (TE), 184 ms; repetition time (TR), 2300 ms; acceleration factor, 2.5; voxel size (acquisition), 0.65 × 0.65 × 1.0 mm^3^; slice number, 180; acquisition time, 6.03 min; and (2) a 3D isotropic fast field echo (FFE) T1-weighted black bone sequence with the following parameters: acquisition time, 5:31 min; acquisition voxel size, 0.43 × 0.43 × 0.43 mm^3^; TR, 10 ms; TE, 1.75 ms; compressed sense; reduction, 2.3; gap, −0.25 mm; water-fat shift (pix)/bandwidth (Hz), 1503/289. The sequences were acquired in axial orientation and reformatted in sagittal and coronal orientation.

All subjects were examined using a 3-Tesla MRI scanner (Ingenia; Philips Healthcare, Best, The Netherlands) with a dedicated 16-channel head, neck, and spine coil (dStream Head Neck Spine coil, Philips Healthcare, Best, The Netherlands). A 3D short tau inversion recovery (STIR) sequence was acquired with the following parameters: echo time, 184 ms; repetition time, 2300 ms; compressed sensitivity encoding (compressed SENSE) with reduction factor of 2.5; voxel size (acquisition), 0.65 × 0.65 × 1.0 mm^3^; slice number, 180, acquisition time, 6.03 min; the sequence was acquired in axial orientation and reformatted in sagittal and coronal orientation.

### OPT and dental radiography

All subjects were examined using a two-dimensional (2D) X-ray device (Orthopos S 2D; Dentsply Sirona, Charlotte, NC, USA). The exposure time for the OPT was set for 14.1 s with the following further settings: 63 kV, 8 mA, and FDP 91. Periapical radiographs were made with an intraoral X-ray unit (Heliodent DS; Dentsply Sirona, Charlotte, NC, USA) with an exposure time between 0.03 and 0.06 s depending on the examined tooth. Further settings were as follows: 60mA, 7 mA, and FDP 9-12. Image acquisition was performed using the paralleling technique in which the film is placed parallel to the long axis of the tooth.

### Image analysis

The 3D STIR sequences were assessed for the occurrence and extent of bone changes associated with AP including edematous changes to the alveolar bone, periradicular cysts, and dental granulomas. The 3D STIR sequences were reconstructed in three orientations (transversal, sagittal, and coronal) and the maximal extent of the bone edema was measured in millimeters. OPTs and periapical radiographs, if available, were assessed for corresponding periapical radiolucencies using a modified periapical index (PAI) score ranging from 1 – healthy to 5 – severe periapical osteolysis with exacerbating features derived from CBCT according to Gürhan et al. [22]. Furthermore, the maximal extent of the periapical radiolucency was measured.

All image analyses (MRI, periapical radiographs and OPTs) were performed by a radiologist (rater 1, MD with 4 years of experience) and by a dentist and radiologist (rater 2, MD, DMD with 7 years of radiological and 2 years of oral surgery experience). In case of severe artifacts due to metallic restorations or movement artifacts, single teeth were excluded from further analysis. The images were rated individually and independently in random order and blinded to clinical or other diagnostic information. Image analyses were performed on a picture archiving and communication system (PACS) workstation certified for clinical use (IDS7 21.2; Sectra, Linköping, Sweden). The MRI and OPT images were read with an interval of at least 8 weeks in between readings, respectively. For intra-reader agreement, 10 patients were assessed once again after 8 weeks by both raters. A standard five-point Likert scale (1=poor, 2=below average, 3=fair, 4=good, 5=excellent) was used for grading of the diagnostic confidence, as well as the overall image quality. The visibility of AP and the radiolucency on OPT and dental radiograph as well as the bone edema on MRI were also graded using a five-point Likert scale based on the extent of the partial volume effect, blurring, image noise, signal inhomogeneity and discrimination from adjacent structures [23].

### Statistics

Findings between the modalities were assessed using Wilcoxon signed-rank tests. Inter- and intra-reader agreements were evaluated with weighted Cohen’s κ for ordinal data and intraclass correlation coefficient (ICC) for nominal measurements. Descriptive statistics were performed using paired t-tests (for numeric variables) and McNemar’s tests (for binary categorical variables). All statistical tests were performed two-sided and a level of significance (α) of 0.05 was used. The data were analyzed using IBM SPSS Statistics for Windows (version 27.0; IBM Corp., Armonk, NY, USA).

## Results

### Patient characteristics and API scores

**Fig. 1:**
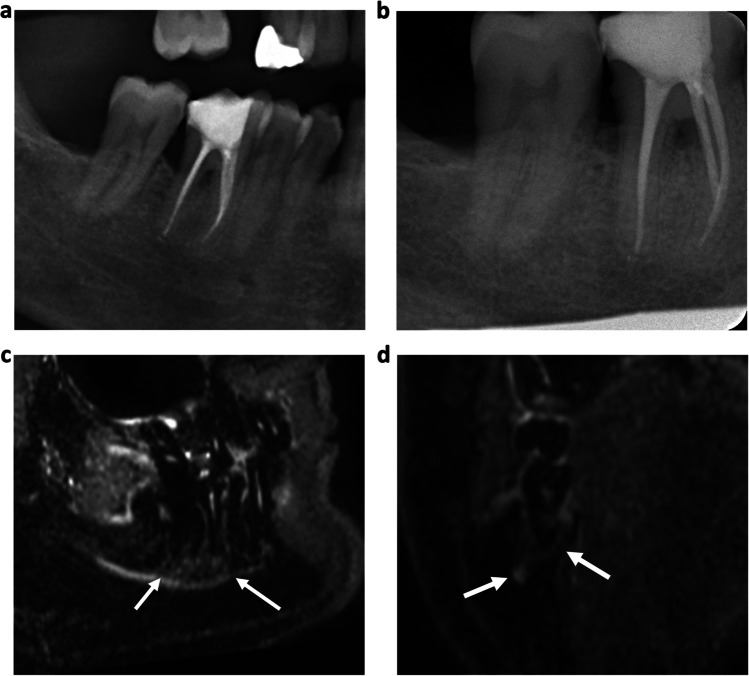
64-year-old patient with known periodontitis. **a** OPT and **b** dental radiograph of the tooth 46 after root canal treatment showing no distinct radiolucency. **c** Sagittal and **d** coronal reconstruction of a 3D STIR sequence showing a bright edema periapical around the root of the treated tooth. The alveolar bone edema on MRI (white arrows) indicates an inflammatory process but might be also associated with physiological changes. Therefore, findings have to be evaluated together with the results from patient anamnesis and clinical examination

**Fig. 2:**
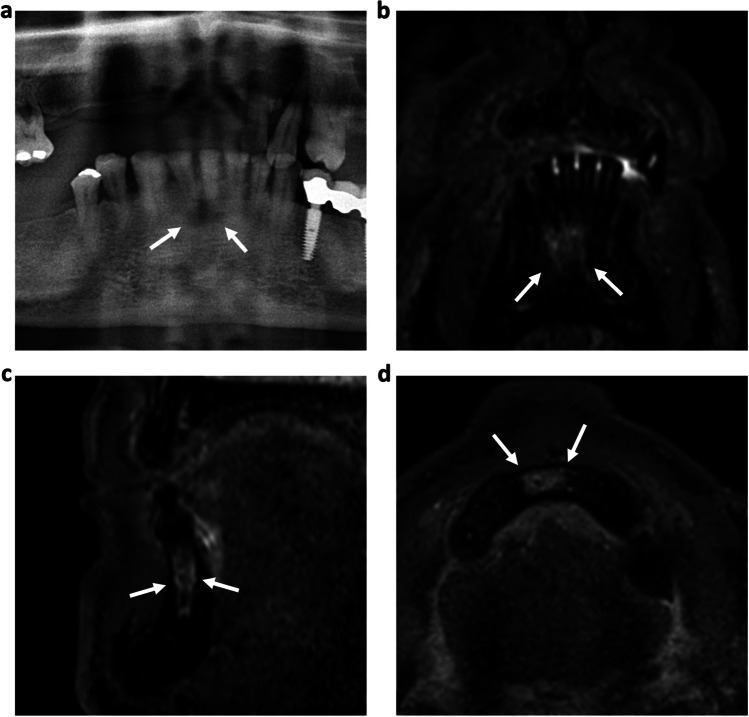
53-year-old patient with symptomatic periodontitis of the tooth 41. A distinctive periapical radiolucency (white arrows) can be seen on OPT (**a**) and dental radiograph (**b**). The coronal (**c**) and axial (**d**) reconstructions of a 3D STIR sequence show a larger bone marrow edema around the periapical lesion, indicating a larger inflammatory reaction (white arrows)

In total, 232 teeth of 37 patients (mean age 62 ± 13.9 years, 18 women) were assessed. Of those, 84 teeth (36%) have been endodontically treated and 148 teeth (64%) have been included without prior treatment. In 174 cases (75%), a reactive bone edema was detected on MRI by both raters (κ 1.00, 95% confidence interval 1.00–1.00) with 69 cases (30%) showing a corresponding radiolucency on OPT (Figs. 1 and 2). In none of the cases, a radiolucency was detected on the OPT without a related bone edema on MRI.

The overall PAI scores measured on the 3D STIR images were significantly higher compared to the PAI scores measured on OPT (rater 1 STIR 2.0 ± 0.3, OPT 1.1 ± 0.7, *P* = 0.02; rater 2 STIR 1.9 ± 1.0, OPT 1.3 ± 0.8, *P* = 0.02, Table 1). Regarding the extent of the bone edema measured on MRI and the corresponding radiolucencies on the OPT, a significantly larger extent was measured on MRI by both raters (rater 1 STIR 2.4 ± 1.6 mm, OPT 0.7 ± 1.1 mm, *P= 0.01*; rater 2 STIR 2.5 ± 1.4 mm, OPT 0.8 ± 1.2 mm, *P= 0.02*). Periapical radiographs were only available in 81 cases (35%), and compared to the 3D STIR, the extent of the bone edema as well as the PAI scores was significantly smaller (bone edema rater 1 STIR 2.4 ± 1.6 mm, dental radiograph 1.1 ± 1.0 mm, *P* = 0.03; rater 2 STIR 2.5 ± 1.4 mm, dental radiograph 1.4 ± 1.3 mm, *P=0.02*; API score rater 1 STIR 2.0 ± 0.3, dental radiograph 1.3 ± 0.4, *P* = 0.03; rater 2 STIR 1.9 ± 1.0, dental radiograph 1.2 ± 0.6, *P* = 0.02)*.*

**Table 1:** API and edema measurements on MRI and OPT

	OPT	Dental radiograph	3D STIR	*p*-value
Mean extent of radiolucency and bone edema (mm)	0.8±1.1	1.3±1.2	2.4±1.4	0.02
Mean PAI scores	1.2±0.7	1.3±0.5	1.9±0.7	0.03

Data are presented as means ± standard deviations

PAI score OPT and dental radiograph (1= healthy, 5 = severe periapical osteolysis), PAI score MRI (0 = healthy, 5 = diameter of periapical radiolucency > 8 mm)

### PAI scores and edema on endodontically and non-treated teeth

The overall PAI scores measured on the 3D STIR images of the endodontically treated teeth were significantly higher compared to the PAI scores measured on OPT and periapical radiographs (rater 1 STIR 1.9 ± 0.7, dental radiograph 1.2 ± 0.3, OPT 1.1 ± 0.2, *P* = 0.01; rater 2 STIR 1.8 ± 0.8, dental radiograph 1.1 ± 0.4, OPT 1.1 ± 0.3, *P* = 0.01). Similar results were obtained for the non-treated teeth (rater 1 STIR 2.3 ± 0.7, dental radiograph 1.3 ± 0.7, OPT 1.2 ± 0.9, *P* = 0.04; rater 2 STIR 2.0 ± 0.9, dental radiograph 1.2 ± 0.5, OPT 1.1 ± 0.5, *P* = 0.03).

**Fig. 3:**
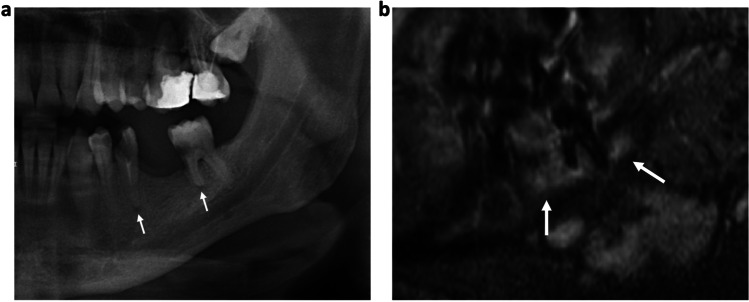
**a** OPT of a 66-year-old patient with a carious lesion and periodontal disease of the tooth 36. Note the small periapical lesion on the conventional OPT (white arrow). On the sagittal reconstruction of the 3D STIR sequence (**b**), an extensive alveolar bone marrow edema (arrows) is detected which might indicate a markedly larger extent of the inflammation. Compared to MRI, the extent of inflammatory reaction may be underestimated by conventional OPT

The overall extent of the bone edema of the endodontically treated teeth measured on MRI was also significantly larger compared to the radiolucencies measured on OPT and periapical radiographs (rater 1 mean STIR 2.2 ± 0.6 mm, dental radiograph 1.1 ± 0.5 mm, OPT 0.9 ± 0.9 mm, *P* = 0.04; rater 2 mean STIR 2.3 ± 0.9 mm, dental radiograph 1.2 ± 0.3 mm, OPT 0.7 ± 2.3 mm, *P* = 0.03), which was similar for the non-treated teeth (rater 1 STIR 2.6 ± 1.2 mm, dental radiograph 1.2 ± 0.7 mm, OPT 1.1 ± 0.9 mm, *P* = 0.01; rater 2 STIR 2.2 ± 0.9 mm, dental radiograph 1.0 ± 0.8 mm, OPT 0.9 ± 1.1 mm, *P* = 0.01) (Fig. 3)*.*

### Visibility of the apical periodontitis and bone edema and diagnostic confidence

The overall image quality of the 3D STIR images as well as OPT images was rated equally good with no significant differences (rater 1 STIR 4.1 ± 0.6, dental radiograph 3.8 ± 0.6, OPT 3.6 ± 0.4, *P* = 0.32; rater 2 STIR 4.2 ± 0.4, dental radiograph 3.9 ± 0.2, OPT 3.8 ± 0.2, *P* = 0.66, Table 2). The diagnostic confidence was overall good for both modalities with no significant differences (rater 1 STIR 3.9 ± 0.4, dental radiograph 3.8 ± 0.5, OPT 3.7 ± 0.3, *P* = 0.56; rater 2 STIR 4.1 ± 0.3, dental radiograph 3.9 ± 0.2, OPT 3.9 ± 0.2, *P* = 0.76).

**Table 2:** Qualitative assessments of MRI and OPT

	Reader 1				Reader 2			
	OPT	Dental radiograph	3D STIR	*p*-value	OPT	Dental radiograph	3D STIR	*p*-value
Visibility of radiolucency and bone edema	2.9±0.2	2.7±0.3	4.8±0.7	0.01	3.0±0.2	2.9±0.4	4.7±0.6	0.01
Visibility of periapical lesions	3.2±0.5	3.5±0.1	4.1±0.3	0.01	3.0±0.2	3.1±0.4	3.9±0.3	0.02
Diagnostic confidence	3.7±0.3	3.8±0.5	3.9±0.4	0.56	3.9±0.2	4.0±0.5	4.1±0.3	0.76
Overall image quality	3.6±0.4	3.8±0.6	4.1±0.6	0.32	3.8±0.4	3.9±0.2	4.2±0.2	0.66

Data are presented as means ± standard deviations

5-point Likert scale (5 = best; 1 = worst)

The visibility of the bone edema was rated significantly better on MRI compared to radiolucency on OPT (rater 1 STIR 4.8 ± 0.7, dental radiograph 2.7 ± 0.3, OPT 2.9 ± 0.2, *P = 0.01*; rater 2 STIR 4.7 ± 0.6, dental radiograph 2.9 ± 0.4, OPT 3.0 ± 0.2, *P = 0.03*). MRI was also rated significantly higher regarding the visibility of AP compared to OPT (rater 1 STIR 4.1 ± 0.3, dental radiograph 3.5 ± 0.1, OPT 3.2 ± 0.5, *P* = 0.01; rater 2 STIR 3.9 ± 0.3, dental radiograph 3.1 ± 0.4, OPT 3.0 ± 0.2, *P* = 0.02).

### Inter- and intra-reader agreement

The inter-reader agreement for the PAI scores measured on the OPT, periapical radiographs, and MRI was substantial to almost perfect (PAI scores OPT: κ 0.95, 95% confidence interval 0.93–1.00; dental radiograph: κ 0.96, 95% confidence interval 0.92–1.00; STIR: κ 0.97, 95% confidence interval 0.94–1.00). A substantial to almost perfect inter-reader agreement was also found for the bone edema (OPT: ICC 0.87, 95% confidence interval 0.83–1.00; dental radiograph: ICC 0.95, 95% confidence interval 0.89–1.00; STIR: ICC 0.94, 95% confidence interval 0.91–1.00). The inter-reader agreement for the visibility of bone marrow edema and periapical radiolucencies was also substantial to almost perfect (κ 0.95, 95% confidence interval 0.90–1.00). For intra-reader agreement, almost all periapical lesions were once more correctly identified by both readers in the 10 patients that were evaluated for this analysis (κ 0.96, 95% confidence interval 0.94–1.00).

## Discussion

In this study, detection and assessment of periapical bone edema in clinically silent periapical disease was feasible and accurate compared to standard dental imaging. Furthermore, the extent of the bone edema as well as the PAI scores was significantly higher compared to the measurements on OPT and periapical radiographs. In all patients with periapical radiolucencies on OPT, a corresponding bone edema was detected on 3D STIR sequences, and the detected bone edema significantly exceeded the measured radiolucency.

So far, detection of AP is commonly achieved using clinical findings in combination with radiographic imaging [2]. For radiographic examination, 2D radiation-based techniques (i.e., intraoral or panoramic radiography) are almost exclusively employed. Due to superimposition of adjacent osseous structures, quality and diagnostic information of 2D images is limited in many cases. However, CT and CBCT can provide 3D images with excellent visualization of hard tissues including the alveolar bone and teeth, but these modalities fail to depict inflammatory processes directly. Additionally, these methods cannot be used as standard procedures in repeated follow-up examinations due to high radiation doses. In contrast, MRI can visualize tooth-related structures including the periodontal ligament with superior resolution and without ionizing radiation. Furthermore, MRI can also depict soft tissue and intraosseous inflammatory changes already at early stages before periapical osteolysis occurs. In this context MRI might be of benefit with regard to detection of initial signs of inflammation.

Geibel et al. recently showed that the assessment of AP using standard T1 and T2 weighted MRI is feasible and accurate compared to CBCT [24, 25]. Furthermore, lesion characterization into e.g. cysts or granulomas was possible and correlated with histopathological findings [24]. However, so far, the surrounding inflammatory reaction and edematous bone changes associated with the periodontal disease as well as AP were not assessed. Yet, Probst et al. were able to show that alveolar bone edema in patients with periodontal disease is associated with the pocket depth in particular over 3 mm and might represent a surrogate marker for the early stages of inflammation before irreversible bone loss has occurred [20]. Yet, they did not assess and compare the extent of the periapical edema and lesions measured on OPT and MRI as well as the effect of endodontic treatment [20]. The magnitude of inflammation is clearly underestimated on conventional dental radiography and OPT [26]. Being able to assess the extent of the osseous inflammation before irreversible bone loss has occurred might improve diagnosis in cases with yet unexplainable persistent odontogenic chronic pain. Moreover, it also might give the physician a better understanding of the time frame in which tooth maintenance or repair has to be achieved.

**Fig. 4:**
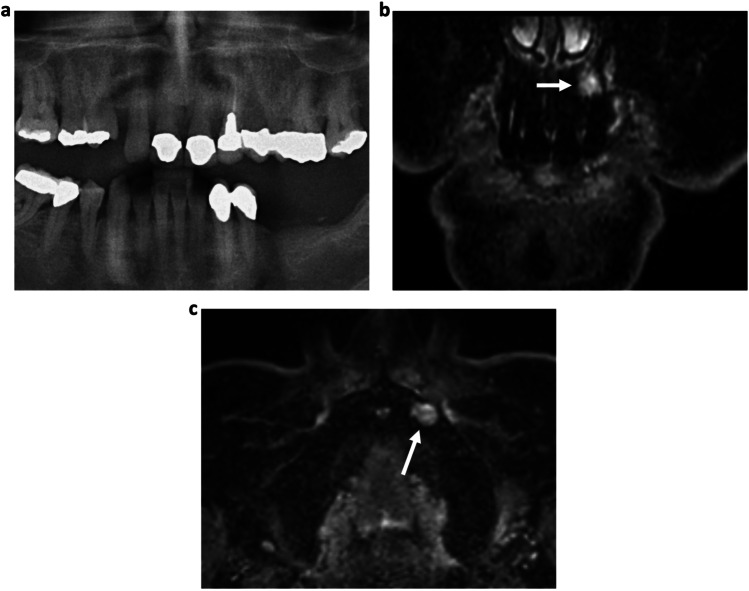
**a** OPT of a 67-year-old patient with root canal treatment of the tooth 21. Note that the roots of the teeth 12 to 21 are not assessable due to superimposition of the nasal cavity and sinuses. On the coronal (**b**) and axial (**c**) reconstructions of the 3D STIR sequence, a distinct periapical cyst/abscess (white arrows) can be detected next to the root of the tooth 21, which would have been missed on conventional radiography

**Fig. 5:**
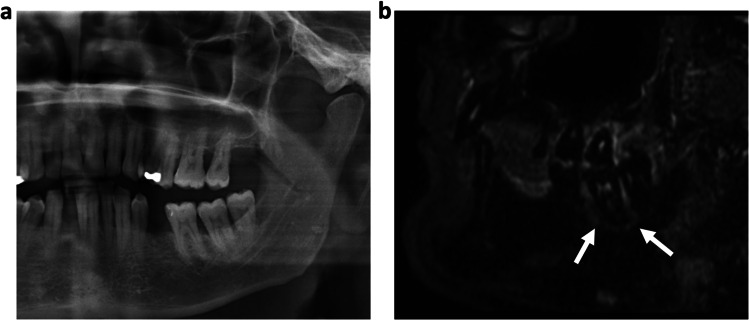
**a** OPT of a non-symptomatic 58-year-old patient. There are no periapical radiolucencies detected on conventional OPT. In contrast, the sagittal reconstruction of the 3D STIR sequence (**b**) shows a bright periapical bone marrow edema around the root of the tooth 37, indicating the formation of an inflammatory complex that has contact to the adjacent roots (white arrows). The missing radiolucency in the OPT may indicate that it is an early-stage inflammation without osteolysis of the alveolar bone

Furthermore, MRI enables the detection of clinically silent, early stage AP in untreated teeth where no irreversible osteolysis has occurred yet and, therefore, no radiolucency could be detected on OPT (Fig. 4). Also, the extent of the inflammation could be estimated more realistically, which may lead to more adequate therapy. In endodontically treated teeth, MRI enables monitoring and evaluation of the therapy success. In endodontics, MRI could be used for visualization of chronic inflammation in the form of persistent edematous changes of the bone. After endodontical treatment, a certain risk for bacterial spread over the pulp cavity to the mandibular bone marrow is given as working length of tooth files are chosen to match the apex and over-instrumentation is not performed in daily routine. In these cases, initial or chronic inflammation, which could cause undulating symptoms, could be detected using water-sensitive MRI sequences. Additionally, in contrast to CBCT and OPT, MRI may further allow for differentiation of AP into cysts, granulomas, or abscesses (Fig. 5). Therefore, MRI could represent a suitable radiation-free tool for early detection of inflammatory changes as well as for potential short-interval therapy monitoring after endodontic instrumentation.

The aim of this study was not to prove technical superiority of a 3D imaging modality compared to 2D projection radiography. The greater accuracy for cross-sectional imaging like MRI, CBCT, and CT has been proven for several clinical settings before [3, 24, 27]. However, the future potential of MRI for dental use has also been shown and summarized in a recent review by Fluegge et al. [28]. In this context, our results emphasize an expanding indication spectrum for MRI in dentistry for capturing otherwise concealed pathophysiological changes of yet unknown significance. With regard to future trends of AI-based reconstruction and associated scan time reduction and prospective increasing availability of dental dedicated MRI for clinical use, further prospective studies are needed to investigate on this modality and compare it to diagnostic gold standards.

Furthermore, the comparison of MRI using bone-specific sequences like the UTE or ZTE and water-sensitive sequences like the STIR-sequence and CBCT as the current gold standard of visualization of complex dental and periapical anatomy would be of clinical interest. Subsequent prospective studies might further pursue the presented idea of generating complementary diagnostic information in context of endodontic pathology detection and therapy monitoring using MRI compared to CBCT.

There are certain limitations to this study which need to be addressed. First of all, in only one-third of the included patients, a dental radiograph as the current imaging gold standard was available. However, this fact can be explained, as all included patients were asymptomatic with regard to percussion and sensitivity testing. Second, the patient cohort was relatively small and heterogeneous, including patients with and without endodontically treated teeth. Focusing on non-treated teeth might increase the reliability of the results. Another limitation is the prolonged examination time and increased costs of MRI compared to conventional dental radiography, which might be difficult to implement in clinical routine but would reduce radiation exposure to the patient. Evaluation of multi-planar MRI also needs more time and experience compared to the evaluation of OPT and dental radiography, which might limit the use to certain specialized centers, where dentists as well as radiologists work closely together as an interdisciplinary team. Furthermore, considering the used voxel size for MRI, there are partial volume effects which could distort the calculated results to a certain extent, as the spatial resolution of x-ray-based imaging cannot be achieved. No further modalities were available (e.g., histopathological samples) as an external standard of reference, nor were follow-up examinations available to assess the development of bone edema over time. To improve the diagnostic value of MRI, further information on associations between specific histopathological changes and intraosseous edema within the tooth-supporting bone should be obtained. Osseous edema as depicted with STIR sequences is associated histologically with the replacement of bone marrow fat by an inflammatory infiltrate in rheumatoid arthritis [29]. Apart from primary inflammatory conditions (i.e., rheumatoid arthritis or spondyloarthritis), also excessive functional stress might cause osseous edema, which needs to be examined in future studies.

Finally, the high sensitivity of MRI in detecting periapical lesions might not only lead to the detection of lesions that would have been missed on conventional methods but might also lead to the detection of lesions which can be considered temporary and might be self-resolving without any additional treatment needed. In order to avoid unnecessary treatment initiation or overtreatment, detected lesions should always be evaluated together with the results from patient anamnesis and clinical examination.

In summary, we conclude that the early detection and assessment of edematous bone changes of AP using 3D STIR imaging was feasible, while the extent of bone edema measured on MRI exceeded the radiolucencies measured on OPT. In clinical routine, dental MRI might be useful for early detection and assessment of AP before irreversible bone loss can be detected by conventional panoramic and periapical radiographs.

## Data Availability

The data are available from the authors upon reasonable request.

## Abbreviations

2D: Two-dimensional
3D: Three-dimensional
AP: Apical periodontitis
CBCT: Cone beam computed tomography
CT: Computed tomography
FFE: Fast field echo
ICC: Intraclass correlation coefficient
MD: Mean difference
MRI: Magnetic resonance imaging
OPT: Conventional panoramic radiography
PACS: Picture archiving and communication system
PAI: Periapical index
STIR: Short tau inversion recovery
TE: Echo time
TR: Repetition time
